# Mitochondria as Epigenetic Regulators of β-Cell Identity and Plasticity: A Metabolo-Epigenetic Perspective

**DOI:** 10.3390/cells15070595

**Published:** 2026-03-27

**Authors:** YongKyung Kim

**Affiliations:** Institute of Medical Science, College of Medicine, Yeungnam University, Daegu 42415, Republic of Korea; yongkyung.kim@yu.ac.kr

**Keywords:** metabolo-epigenetics, β-cell dedifferentiation, epigenic rejuvenation

## Abstract

The progressive decline in functional β-cell mass in Type 2 Diabetes (T2D) is increasingly recognized not as a simple apoptotic loss, but as a complex erosion of cellular identity termed “dedifferentiation.” Central to this phenotypic shift is the metabolo-epigenetic axis, where mitochondria act as the primary sensing hub, transducing nutrient flux into biochemical signals that govern the chromatin landscape. This review synthesizes current evidence on how mitochondrial metabolites—including Acetyl-CoA, α-ketoglutarate, and NAD+—serve as obligatory co-factors for the epigenetic machinery. We explore how chronic metabolic stress triggers a “Systemic epigenetic destabilization,” leading to the loss of lineage-specific markers and the formation of persistent “metabolic scars.” Furthermore, we discuss the clinical implications of these changes, specifically regarding the phenomenon of metabolic memory and the molecular limits of β-cell reversibility. By integrating foundational transcriptional studies with emerging epigenomic data, we propose that targeting the mitochondrial–epigenetic axis offers a strategic window for re-differentiating failing β-cells and restoring glycemic homeostasis.

## 1. Introduction: The Evolution of β-Cell Failure—From Apoptosis to Dedifferentiation

The pathogenesis of Type 2 Diabetes (T2D) has long been characterized by a progressive and seemingly inexorable decline in functional β-cell mass. For decades, the prevailing consensus—driven by seminal histological observations [1]—was that chronic nutrient overload triggers a terminal apoptotic cascade. However, this “death-centric” model has undergone a profound re-evaluation. A landmark shift occurred with the work of Talchai et al. [2], which utilized lineage-tracing models to demonstrate that failing β-cells do not necessarily vanish; instead, they lose their specialized identity and regress to a progenitor-like state—a process termed “dedifferentiation.” This discovery reframed β-cell failure as a loss of cellular maturity, a phenomenon subsequently confirmed in human T2D islets [3]. Recent advances in high-resolution multi-omics have added layers of complexity to this phenotypic plasticity. Single-cell RNA sequencing (scRNA-seq) studies by Baron et al. and Ahlqvist et al. [4,5] have revealed that β-cell dedifferentiation is not a uniform event but a highly heterogeneous process.

While some cells revert to an *Ngn3+* state, others undergo trans-differentiation, adopting features of other islet cell types like α- or δ-cells [6]. A fundamental requirement for preventing this regression is the maintenance of a core transcriptional network that locks the cell in a functional state. In this regard, the landmark study from the Lori Sussel laboratory [7] demonstrated that *NeuroD1* is indispensable not only for the achievement of β-cell maturity but also for its continuous maintenance. Their work revealed that the loss of *NeuroD1* leads to a rapid decline in the expression of insulin and other key identity markers like *MafA* and *Nkx6.1*, effectively pushing the cell toward a dysfunctional state. Metabolo-epigenetic axis as a central governing force in β-cell biology. Mitochondria, as the primary “metabolic sensing hub,” facilitate this by generating intermediary metabolites—specifically Acetyl-CoA, α-ketoglutarate (α-KG), and NAD^+^, which function as obligatory cofactors for chromatin-modifying enzymes [8].

The chromatin landscape is often disrupted in the diabetic state, a process accompanied by aberrant mitochondrial dynamics and fragmentation [9]. This metabolic stagnation leads to a shift in the availability of these cofactors, resulting in systemic epigenetic destabilization where the repressive and active marks maintaining the β-cell-specific gene program are altered. Integrating these metabolic cues with protective signalling pathways is essential for maintaining β-cell resilience.

In this context, the role of intracellular phosphatases has emerged as a critical safeguard. As a representative example of homeostatic regulators, [10] has identified PTPN2 (Protein Tyrosine Phosphatase Non-receptor type 2) as a vital molecular shield against inflammatory stress. We demonstrated that PTPN2 deficiency in islet β-cells significantly exacerbates inflammation-induced dysfunction and compromises cellular viability. These findings highlight the PTPN2-mitochondrial axis as a key protective pathway as a key homeostatic regulator that preserves β-cell integrity, suggesting that its loss sensitizes the cell to the toxic synergistic effects of metabolic and inflammatory chronic stress.

While previous reviews [11,12,13,14] have extensively catalogued epigenetic marks in T2D, this manuscript provides a distinct perspective by focusing on the spatiotemporal coupling between mitochondrial bioenergetics and chromatin architecture. We emphasize how the fluctuating metabolic flux—rather than static metabolite levels—serves as a dynamic rheostat for β-cell identity, offering a novel framework for understanding the transition from functional plasticity to epigenetic rigidity.

**Figure 1:**
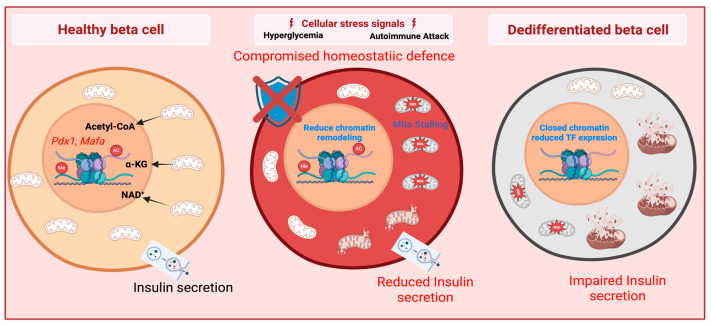
Graphical representation of the transition from a functional β-cell to a dedifferentiated state via the metabolo-epigenetic axis. Created in BioRender. Kim, Y. (2026) https://BioRender.com/r0i9zjg.

This review aims to synthesize the emerging evidence linking mitochondrial bioenergetics and the epigenetic landscape of the β-cell. By exploring how “metabolic scars” contribute to the clinical phenomenon of metabolic memory [15], we propose that the metabolo-epigenetic axis represents a strategic therapeutic window to preserve and restore functional β-cell mass in T2D (Figure 1). For a more comprehensive discussion on mitochondrial–epigenetic crosstalk, readers are referred to several excellent reviews published within the past five years, including those focused specifically on β-cell mitochondrial metabolism [16] and broader metabolic–epigenetic interactions [17,18,19,20,21].

(Left) Healthy Cell: Under physiological conditions, robust mitochondrial metabolism generates key co-factors such as Acetyl-CoA, α-KG, and NAD+. These metabolites act as essential substrates for epigenetic enzymes (e.g., histone acetyltransferases and demethylases), maintaining an open chromatin landscape at lineage-defining gene loci like *Pdx1* and *Mafa*. This epigenetic fidelity ensures sustained insulin synthesis and secretion. (Middle) Cellular Stress and Failed Defence: Chronic exposure to hyperglycaemia and autoimmune-driven inflammatory signals (e.g., cytokines) induces mitochondrial stalling and excessive ROS production. A “Compromised homeostatic defense”—exemplified by the loss of internal regulators like PTPN2—exacerbates this process by leaving the cell vulnerable to inflammatory “second hits.” This results in a reduced chromatin remodelling capacity and impaired insulin secretion. (Right) Dedifferentiated β-cell: umulative systemic epigenetic destabilization leads to a closed chromatin state, characterized by the decommissioning of key enhancers and reduced expression of maturation transcription factors (TFs). The resulting lineage erosion leads to impaired insulin output and a loss of functional β-cell identity, a hallmark of advanced Type 2 Diabetes.

## 2. Mitochondrial Metabolites as Epigenetic Modifiers: The Biochemical Language of Chromatin

The concept of “Metabolo-epigenetics” posits that the epigenome acts as a dynamic interface between the cellular environment and the genome. In pancreatic β-cells, mitochondria are not merely ATP generators; they function as a sophisticated “metabolic sensing hub” that dictates the availability of the biochemical co-substrates required for chromatin-modifying enzymes [8,22].

### 2.1. From Glycolysis to Mitochondria: The Glucose-Driven Epigenetic Blueprint

The epigenetic landscape of the β-cell is fundamentally primed by the rate of glucose oxidation. In mature β-cells, glucose enters through high-capacity transporters (GLUT1/2) and is immediately phosphorylated by Glucokinase (GCK), the rate-limiting “glucose sensor” [23]. Unlike other cell types, β-cells exhibit high glycolytic flux coupled with low lactate dehydrogenase (LDH) activity, ensuring that the majority of glucose-derived pyruvate is shunted into the mitochondria [24,25]. This glycolytic-to-mitochondrial flux is not just for fuel; it partially regulates β-cell function through enduring epigenomic changes.

### 2.2. Acetyl-CoA: The Metabolic Signal for Histone Acetylation

Acetyl-CoA stands at the nexus of glucose oxidation and epigenetic control. Glucose-derived pyruvate enters the mitochondria and is converted by the Pyruvate Dehydrogenase Complex (PDC) into Acetyl-CoA, which enters the TCA cycle to form citrate. In β-cells, this citrate is exported via the mitochondrial citrate carrier (CIC) and converted back into nucleocytosolic Acetyl-CoA by ATP-citrate lyase (ACLY) [26]. This pool of Acetyl-CoA is the primary donor for HAT-mediated histone acetylation. High glucose promotes ACLY phosphorylation and activity, maintaining H3 and H4 acetylation at the promoters of *Ins1/2*, *Pdx1,* and *MafA* [26,27]. Conversely, mitochondrial dysfunction leads to reduced citrate export and a precipitous drop in HAT activity [22], often coupled with the impairment of AMPK-mediated regulation, further exacerbating the loss of active marks that define the mature β-cell identity [3,28]. Beyond glucose, mitochondrial oxidation of fatty acids has also been shown to generate acetyl-CoA that serves as a substrate for histone acetylation [29]. The resulting acetyl-CoA is thought to shuttle between the mitochondria and nucleus via the acetyl-carnitine shuttle [30]. Furthermore, recent work using subcellular metabolomics and isotope tracing has demonstrated that the mitochondrial catabolism of branched-chain amino acids (BCAAs) generates propionyl-CoA, which serves as a substrate for histone lysine propionylation—a mechanism observed across multiple cell types including pancreatic cancer cells [31].

### 2.3. α-Ketoglutarate (α-KG): The Switch for DNA and Histone Demethylation

The TCA cycle intermediate α-KG is an obligatory co-substrate for the Fe(II)-dependent dioxygenase superfamily, including the JmjC histone demethylases and the TET DNA demethylases [32]. These enzymes are highly sensitive to the α-KG/succinate ratio; succinate and fumarate act as competitive inhibitors, leading to an “oncometabolite” effect that promotes hypermethylation [32,33]. In failing β-cells, the imbalance in mitochondrial oxidation results in a decreased α-KG/succinate ratio, which compromises the demethylation of H3K9 and H3K27, thereby locking enhancers in a repressed state [34,35]. This epigenetic “stiffness” contributes significantly to the phenomenon of metabolic memory [36].

### 2.4. NAD+ and Sirtuins: Redox-Coupled Deacetylation

The oxidized form of nicotinamide adenine dinucleotide (NAD+) is the essential cofactor for Class III histone deacetylases, Sirtuins (SIRT1-7) [37]. SIRT1 regulates β-cell function by deacetylating Pgc-1α and FoxO1, enhancing mitochondrial biogenesis and stress resistance [38,39]. Chronic hyperglycaemia induces a state of “pseudohypoxia,” where the NADH/NAD+ ratio increases, limiting SIRT1 activity [40]. This impairment results in the hyperacetylation of non-histone proteins and the loss of mitochondrial quality control, forming a vicious cycle of metabolic and epigenetic decay [41,42]. Hyperacetylation of non-histone proteins, which can occur in both the nucleus and mitochondria, is a key feature of metabolic stress. It is important to emphasize that, unlike in the nucleus where protein acylation is enzyme-catalysed and reaches high stoichiometry, mitochondrial protein acylation largely occurs nonenzymatically at low stoichiometry. Its regulatory significance, including its role in β-cell acetylation, remains an emerging area of investigation [43,44].

### 2.5. One-Carbon Metabolism and SAM: The Reservoir of Methylation

Mitochondrial one-carbon metabolism facilitates the production of S-adenosylmethionine (SAM), the universal methyl donor for DNA and histone methyltransferases (HMTs) [45]. The mitochondrial folate cycle and the methionine cycle must be synchronized to maintain the SAM/SAH ratio [46]. Disruption of this flux in T2D leads to a shortage of methyl groups that normally maintain the silencing of “disallowed genes” such as *Ldha* and *Hk1* [24,25]. The erosion of repressive marks like H3K27me3 allows for the aberrant activation of foetal gene programs, a hallmark of dedifferentiation [47,48,49].

## 3. Mitochondrial Dysfunction and the Systemic Epigenetic Destabilization in T2D

In the progression of Type 2 Diabetes (T2D), the β-cell undergoes a transition from metabolic compensation to a state of terminal failure. This “metabolic exhaustion” is fundamentally a mitochondrial crisis. When the mitochondrial workload exceeds its capacity, the organelle’s role shifts from a provider of regulatory metabolites to a driver of epigenetic destabilization. This “Systemic epigenetic destabilization” is not a random erosion but a targeted silencing of the mature β-cell gene program, mediated by the failure of mitochondrial homeostatic signalling [25,50].

### 3.1. Mitochondrial Dynamics and the Structural Basis of Epigenetic Drift

Mitochondria in healthy β-cells exist in a dynamic, reticulated network maintained by a precise balance of fusion and fission [9]. Chronic glucolipotoxicity disrupts this balance, shifting the morphology toward fragmented, dysfunctional globoids via the upregulation of *Drp1* and the loss of *Opa1* and *Mfn2* [51,52,53]. This structural fragmentation is not merely a byproduct of stress; it directly impairs the efficient production and transport of citrate and α-KG to the nucleus. As demonstrated by Sidarala et al. [53], impaired mitochondrial fusion leads to the loss of mitochondrial DNA (mtDNA) integrity and a subsequent decline in the oxidative phosphorylation (OXPHOS) capacity, which effectively starves the epigenetic machinery of its obligatory cofactors, leading to a global loss of the active H3K27ac mark at β-cell enhancers.

### 3.2. Oxidative Stress and the Inhibition of Demethylases

The accumulation of Reactive Oxygen Species (ROS) is a hallmark of mitochondrial failure in T2D. While physiological levels of ROS serve as signalling molecules, excessive superoxide production from the electron transport chain (ETC) leads to the oxidative inactivation of iron–sulphur cluster-containing enzymes [54]. Crucially, the TET DNA demethylases and JmjC histone demethylases require Fe(II) as a cofactor. Mitochondrial ROS can oxidize Fe(II) to Fe(III), effectively inhibiting these enzymes and leading to DNA hypermethylation at critical loci such as the *Pdx1* promoter [54,55]. Furthermore, work by Schofield and Ratcliffe [56] has shown that oxidative stress can lead to the “pseudohypoxic” stabilization of HIF-1α, which further redirects glucose flux away from the mitochondria toward lactate (the Warburg effect), exacerbating the shortage of mitochondrial co-substrates for chromatin maintenance.

### 3.3. Mitophagy Failure and the Persistence of “Epigenetic Scars”

The quality control of mitochondria through mitophagy is essential for maintaining a healthy pool of organelles capable of precise metabolic signalling. In T2D, PINK1/Parkin pathway is often impaired, leading to the accumulation of “damaged-but-not-cleared” mitochondria that produce suboptimal metabolite ratios [53,57]. These dysfunctional mitochondria contribute to the persistence of “epigenetic scars”—pathogenic chromatin modifications that remain even after normoglycemia is restored [58]. This phenomenon, known as metabolic memory, is driven by the inability of the cell to reset its H3K4me1/H3K4me3 ratio at glucose-responsive enhancers, a process that requires a robust and consistent supply of mitochondrial-derived α-KG [59,60].

### 3.4. The Synergistic Impact of Inflammation and Compromised Homeostatic Defence

As we have recently highlighted, the metabolic collapse is further accelerated by inflammatory signalling. The loss of homeostatic safeguards, such as *PTPN2*, exacerbates mitochondrial sensitivity to IFN-γ and TNF-α, leading to a rapid decline in mitochondrial membrane potential [10]. This inflammatory–mitochondrial crosstalk acts as a “second hit” to the epigenome. While metabolic stress stalls the TCA cycle, inflammation-induced ROS further inhibits the salvage of NAD+ through the NAMPT pathway, severely limiting the SIRT1-mediated deacetylation of histones and PGC-1α [61,62]. The convergence of these pathways results in the profound erosion of the β-cell-specific transcriptional circuit, cementing the dedifferentiated state.

## 4. Loss of Lineage-Specific Markers and the Epigenetic Landscape

The terminal stage of β-cell failure is defined not by cell death, but by the profound erosion of the lineage-specific transcriptional program. This process, termed dedifferentiation, is characterized by the down-regulation of “identity genes” and the aberrant de-repression of “disallowed” or “fetal” genes. This phenotypic shifting is fundamentally anchored in a global reconfiguration of the epigenetic landscape, where the loss of mitochondrial-derived regulatory metabolites acts as a catalyst for the dissolution of β-cell-specific enhancers [3,63].

### 4.1. The Dissolution of Core Transcriptional Hubs: PDX1, MAFA, and NKX6.1

The maintenance of β-cell identity depends on a hierarchical network of transcription factors (TFs), primarily *PDX1*, *MAFA*, and *NKX6.1*, which co-occupy thousands of genomic loci to maintain open chromatin states at lineage-specific enhancers [47,64]. In the diabetic environment, mitochondrial insufficiency leads to a reduction in H3K27ac and H3K4me3 marks at these critical hubs. As shown by Ferrer and colleagues [63], the loss of these active marks results in the “closing” of stretch enhancers that drive the expression of insulin secretion machinery. The reduction in PDX1 occupancy, in particular, is a watershed event; its absence leads to the recruitment of corepressors and the establishment of repressive H3K9me3 marks, effectively “locking” the cell out of its mature state [65,66].

### 4.2. Epigenomic De-Repression: The Rise of Disallowed and Progenitor Genes

A hallmark of the dedifferentiated β-cell is the activation of “disallowed genes” (e.g., *Ldha*, *Hk1*, *Mct1*) and progenitor markers (e.g., *Sox9*, *Ngn3*). Under physiological conditions, these genes are strictly silenced by high levels of DNA methylation and H3K27me3, maintained by the consistent supply of mitochondrial SAM and α-KG [67,68]. However, the “metabolic stagnation” in T2D compromises the activity of TET demethylases and Polycomb Repressive Complex 2 (PRC2). The erosion of these repressive epigenetic barriers allows for a “transcriptional leakage,” where the β-cell adopts a hybrid, dysfunctional phenotype that resembles an immature foetal cell [36,69]. The stringent suppression of ‘disallowed genes’ (e.g., *Ldha*, *Hk1*) is a prerequisite for the specialized glucose-sensing role of β-cells. This active repression is primarily orchestrated by the Polycomb Repressive Complex 2 (PRC2), which deposits H3K27me3 marks to “lock” these loci in a transcriptionally silent state. Chronic metabolic stress disrupts EZH2 dynamics (the catalytic subunit of PRC2), leading to the erosion of these repressive barriers and subsequent lineage infidelity.

### 4.3. Enhancer Reprogramming and the “Metabolic Scarring” Effect

The systemic epigenetic destabilization in T2D is not merely transient but involves the formation of permanent “metabolic scars” on the enhancer landscape. Even after the normalization of glucose levels, many β-cell enhancers remain in a decommissioned state [69,70]. This persistence is driven by the failure of the cell to re-establish the H3K4me1/2 “priming” marks, a process that requires robust mitochondrial anaplerosis and metabolic signalling [71,72]. The work of Sandovici et al. [73] further underscores that once these epigenomic programs are disrupted by chronic metabolic flux, the “epigenetic memory” of the failed state can prevent functional recovery, even with intensive insulin therapy.

### 4.4. Convergence with Mitochondrial Homeostatic Safeguards

While the PTPN2-mitochondrial axis acts as a representative shield against inflammatory stress, its deficiency leads to a rapid decline in the stability of the β-cell-specific gene circuit under metabolic overload [74]. In the absence of PTPN2, the synergy between cytokine-induced ROS and mitochondrial stalling creates “Synergistic metabolic insults” for the epigenetic machinery. This results in an accelerated loss of MAFA and NKX6.1 expression, suggesting that such homeostatic signalling pathways are critical prerequisite for the maintenance of the epigenetic barriers that prevent lineage-specific marker loss [75,76]. The key interactions between mitochondrial metabolites and their epigenetic targets are summarized in Table 1.

## 5. Clinical Implications: Reversibility and Metabolic Memory

**Figure 2:**
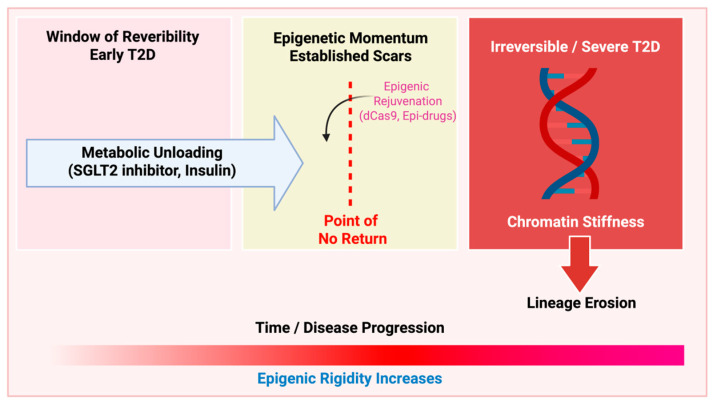
Conceptual model of the progression of β-cell dysfunction and the temporal limits of therapeutic reversibility. Created in BioRender. Kim, Y. (2026) https://BioRender.com/m0l5w4k.

The transition from functional plasticity to epigenetic rigidity defines the clinical progression of T2D (Figure 2). The clinical management of Type 2 Diabetes (T2D) is often confounded by the “legacy effect,” or metabolic memory, wherein a period of early glycaemic instability continues to drive the progression of complications and β-cell failure long after euglycemia is achieved [15,77]. From an epigenetic perspective, this phenomenon represents the persistence of “metabolic scars”—enduring chromatin modifications that fail to reset despite the withdrawal of the initial stressor. Understanding the molecular basis of this memory, and the extent of its reversibility, is paramount for developing next-generation disease-modifying therapies [78,79]. Despite the transformative potential of site-specific epigenetic editing in erasing metabolic scars, several hurdles remain for its clinical translation. The primary barrier is the cell-specific delivery of these molecular tools. Systemic administration of epigenetic modulators poses a significant risk of off-target effects in non-target tissues. Therefore, developing cell-specific delivery systems, such as ligand-conjugated lipid nanoparticles (LNPs) or engineered adeno-associated virus (AAV) vectors, is a critical prerequisite for therapeutic success. Furthermore, defining the “therapeutic window”—the specific time frame during which epigenetic rigidity has not yet become irreversible—will be essential for effective patient stratification.

### 5.1. Molecular Basis of Metabolic Memory in the β-Cell

Metabolic memory is fundamentally encoded through long-lived epigenetic marks, primarily DNA methylation and certain histone lysine methylations (e.g., H3K4me1/3 and H3K9me3). In β-cells exposed to chronic hyperglycaemia, the initial mitochondrial “stalling” leads to an aberrant surge in ROS and a deficit in α-ketoglutarate, which inhibits the TET and JmjC demethylases [60,80]. As demonstrated by El-Osta and colleagues [58], even transient spikes in glucose can induce a persistent increase in H3K4me at pro-inflammatory promoters (e.g., *p65* of NF-κB) that remains for days after returning to normoglycemia. In the context of the β-cell, this “epigenetic momentum” maintains the silencing of identity genes like *Pdx1* and *MafA*, preventing the cell from reclaiming its mature functional state even when metabolic parameters are clinically normalized [36,71].

### 5.2. Reversibility of Dedifferentiation: Hope and Limitations

A pivotal question in diabetes research is whether the dedifferentiated β-cell can be “re-differentiated.” Animal studies using intensive insulin therapy or SGLT2 inhibitors have shown that unloading the β-cell can partially restore the expression of key TFs like NKX6.1 [81,82]. However, human data suggest a more complex reality. While insulin secretion can improve, the “epigenomic landscape” often remains skewed. As highlighted by Sandovici et al. [73], certain glucose-induced methylation marks are remarkably stable, acting as a brake on functional recovery. This suggests a “point of no return” in systemic epigenetic destabilization, where the chromatin becomes too “stiff” for purely metabolic interventions to reverse [47,83]. Due to their widespread clinical use, ongoing and future studies of GLP-1–based treatments for T2D should also consider their potential impact on β-cell epigenetic reprogramming, an emerging area of investigation that has been recently highlighted [84].

### 5.3. Therapeutic Strategies: Beyond Glycaemic Control

To overcome the constraints of metabolic memory, a dual approach targeting both mitochondrial health and epigenetic erasing is required. Emerging therapies such as SGLT2 inhibitors not only lower systemic glucose but may also reduce β-cell workload and mitochondrial ROS, potentially slowing the accumulation of new epigenetic scars [85,86]. Furthermore, the use of “epi-drugs”—small-molecule inhibitors of HATs or HDMs—is being explored to actively erase pathogenic marks [87]. Focusing on homeostatic defence mechanisms, such as *PTPN2* [10] adds another layer to this strategy; by preserving the β-cell’s internal defence against inflammatory “second hits,” we may broaden the window of reversibility, preventing the metabolic collapse from becoming an irreversible lineage erosion [75,82]. In a clinical context, delivering epigenetic editing tools specifically to β-cells without affecting other tissues remains a major barrier. Systemic administration risks global epigenetic dysregulation; therefore, developing β-cell-specific delivery systems, such as ligand-conjugated lipid nanoparticles (LNPs) or engineered viral vectors, is a critical prerequisite for the therapeutic application of “erasing” metabolic scars.

### 5.4. Conclusion: A New Frontier in β-Cell Preservation

In summary, the transition from β-cell dysfunction to permanent failure is a journey through an increasingly rigid epigenetic landscape. The intersection of mitochondrial metabolism and chromatin remodelling provides a mechanistic explanation for why early, intensive glycaemic control is far more effective than late intervention. Future research must focus on identifying the specific “erasers” that can target the metabolic scars of T2D. By integrating mitochondrial anaplerosis with the reinforcement of protective signalling hubs like *PTPN2*, we may finally move beyond managing symptoms to actively restoring the cellular identity of the pancreatic β-cell. Recent findings by Suleiman et al. (2025) demonstrate that T2D β-cells retain significant functional plasticity under euglycemic conditions. However, the persistence of epigenetic alterations in some patients suggests a transition from plasticity to rigidity. This heterogeneity in clinical remission may depend on whether the chromatin landscape has reached a “point of no return,” where purely metabolic restoration can no longer erase established pathogenic “metabolic scars” [88].

## 6. Conclusions and Future Perspectives

### 6.1. Deciphering the “Point of No Return”

A critical challenge for future research is to define the temporal window during which β-cell dedifferentiation remains reversible. While early intervention can restore functional markers, prolonged metabolic stress appears to cement “metabolic scars” that resist conventional glucose-lowering therapies [58,73]. Identifying the specific molecular “thresholds”—such as the irreversible gain of repressive H3K9me3 marks or the terminal loss of stretch enhancer accessibility—will be paramount. Determining these markers will allow clinicians to move beyond systemic glycaemic monitoring toward a more precise, molecularly-driven assessment of β-cell health and recovery potential.

### 6.2. Toward “Epigenetic Rejuvenation”: Challenges and Opportunities

The persistence of metabolic memory remains the most significant hurdle in the long-term management of T2D. The next generation of diabetes research must transition toward epigenetic rejuvenation. This involves a multi-pronged approach: not only normalizing metabolic flux through mitochondrial anaplerosis but also actively “erasing” pathogenic epigenetic marks. Emerging technologies, such as site-specific epigenetic editing tools (e.g., dCas9-based modulators) and small-molecule inhibitors of specific histone methyltransferases, offer the potential to re-establish active chromatin hubs at key loci like *Pdx1* and *MafA* [69,87]. However, achieving cell-type specificity remains a formidable task that requires innovative delivery systems.

### 6.3. Concluding Remarks

In conclusion, the path to reclaiming functional β-cell mass in T2D lies at the intersection of mitochondrial bioenergetics and epigenetic stability. By preserving the mitochondrial “metabolic sensor,” we may be able to extend the window of reversibility and prevent the terminal loss of cellular identity. As we refine our ability to manipulate the metabolo-epigenetic axis, the goal of “re-differentiating” failing β-cells moves closer to clinical reality. This promises a future where diabetes management evolves from symptom control to a proactive restoration of the β-cell’s unique epigenetic and functional blueprint (Figure 2).

Window of Reversibility (Early T2D): During the early stages of Type 2 Diabetes (T2D), β-cell identity remains largely intact. Functional deficits are primarily driven by metabolic overload, which can be reversed through metabolic unloading strategies, such as SGLT2 inhibitors or intensive insulin therapy. At this stage, normalization of glucose levels allows for the partial restoration of maturation transcription factors. Epigenetic Momentum and the Point of No Return: As metabolic and inflammatory stress persist, “metabolic scars” (pathogenic epigenetic marks) begin to stabilize. This phase represents a critical “Point of No Return,” where the endogenous defence mechanisms are overwhelmed. Beyond this threshold, traditional metabolic interventions are insufficient to restore cellular identity. Irreversible systemic epigenetic destabilization (advanced T2D): In advanced T2D, the chromatin landscape reaches a state of high rigidity or “stiffness,” leading to permanent lineage erosion. However, emerging epigenetic rejuvenation tools (e.g., dCas9-based modulators, epi-drugs) offer a novel avenue to bypass this point of no return by actively erasing pathogenic marks and re-establishing active chromatin hubs.

## Figures and Tables

**Table 1:** Summary of mitochondrial metabolites and their epigenetic targets in β-cells.

Metabolite	Primary Source	Epigenetic Enzyme	Epigenetic Change	Target Genes/Loci
Acetyl-CoA	Glucose, FAs	HATs (p300/CBP)	↓ Histone Acetylation	Pdx1, MafA, Ins1/2
A-KG	TCA cycle	TETs, JmjC HDMs	↓ DNA/Histone Demethylation	Ldha, Hk1 (Disallowed)
NAD+	Oxphos	SIRT1, SIRT6	↓ Deacetylation	FoxO1, PGC-1α
SAM	1-Carbon cycle	DNMTs, HMTs	↑ DNA Hypermethylation	Glp1r, Slc2a2 (Glut2)
Propionyl-CoA	BCAAs	HATs	↑ Lysine Propionylation	Chromatin Hubs

## Data Availability

No new data were created or analysed in this study.

## Abbreviations

The following abbreviations are used in this manuscript:

α-KG	α-ketoglutarate
ACLY	ATP-citrate lyase
AAV	Adeno-associated virus
Acetyl-CoA	Acetyl coenzyme A
BCAA	Branched-chain amino acid
CBP	CREB-binding protein
CIC	Mitochondrial citrate carrier (Slc25a1)
DNMT	DNA methyltransferase
dCas9	Dead Cas9
DRP1	Dynamin-related protein 1
EZH2	Enhancer of zeste homolog 2
FA	Fatty acid
FoxO1	Forkhead box protein O1
GCK	Glucokinase
GLP-1	Glucagon-like peptide-1
Glut2	Glucose transporter 2 (Slc2a2)
HATs	Histone acetyltransferases
HDACs	Histone deacetylases
HDMs	Histone demethylases
HIF-1α	Hypoxia-inducible factor 1-alpha
Hk1	Hexokinase 1 (disallowed gene)
HMTs	Histone methyltransferases
Ldha	Lactate dehydrogenase A (disallowed gene)
LNP	Lipid nanoparticle
IFN-γ	Interferon-gamma
JmjC	Jumonji C domain-containing
MAFA	V-maf musculoaponeurotic fibrosarcoma oncogene homolog A
MCT1	Monocarboxylate transporter 1
MFN2	Mitofusin 2
NAD+	Nicotinamide adenine dinucleotide
NeuroD1	Neuronal differentiation 1
NGN3+	Neurogenin 3
NKX6.1	NK6 homeobox 1
OPA1	Optic atrophy 1
PDX1	Pancreatic and duodenal homeobox 1
PGC-1α	Peroxisome proliferator-activated receptor gamma coactivator 1-alpha
PRC2	Polycomb repressive complex 2
PTPN2	Protein tyrosine phosphatase non-receptor type 2
ROS	Reactive oxygen species
SAM	S-adenosylmethionine
SGLT2i	Sodium–glucose cotransporter-2 inhibitors
SIRT	Sirtuin
SOX9	SRY-box transcription factor 9
T2D	Type 2 Diabetes
TF	Transcription factor
TCA cycle	Tricarboxylic acid cycle
TET	Ten-eleven translocation
TNF-α	Tumour necrosis factor-alpha
